# Correlation between steroid levels in follicular fluid and hormone synthesis related substances in its exosomes and embryo quality in patients with polycystic ovary syndrome

**DOI:** 10.1186/s12958-021-00749-6

**Published:** 2021-05-17

**Authors:** Li Yu, Miao Liu, Zhenxin Wang, Te Liu, Suying Liu, Beili Wang, Baishen Pan, Xi Dong, Wei Guo

**Affiliations:** 1 Department of Laboratory Medicine, Zhongshan Hospital, Fudan University, No. 111 Yi Xue Yuan Road, Shanghai, 200032 PR China; 2 Reproductive Medicine Center, Zhongshan Hospital, Fudan University, No. 250 Xiao Mu Qiao Road, Shanghai, 200032 PR China; 3 Shanghai Geriatric Institute of Chinese Medicine, Shanghai University of Traditional Chinese Medicine, No.725 South Wan Ping Road, Shanghai, 200031 PR China; 4 Department of Laboratory Medicine, Xiamen Branch, Zhongshan Hospital, Fudan University, No. 668 Jin Hu Road, Xiamen, 361015 PR China

**Keywords:** Infertility, Polycystic ovary syndrome, Follicular fluid, Estrogen, Progestogen, Exosomes

## Abstract

**Background:**

Polycystic ovary syndrome (PCOS) is an endocrine and metabolic disorder with various manifestations and complex etiology. Follicular fluid (FF) serves as the complex microenvironment for follicular development. However, the correlation between the concentration of steroid in FF and the pathogenesis of PCOS is still unclear.

**Methods:**

Twenty steroid levels in FF from ten patients with PCOS and ten women with male-factor infertility undergoing in vitro fertilization were tested by liquid chromatography-tandem mass spectrometry (LC-MS/MS) in order to explore their possibly correlation with PCOS. Meanwhile, the mRNA levels of core enzymes in steroid synthesis pathway from exosomes of FF were also detected by qPCR.

**Results:**

The estriol (*p* < 0.01), estradiol (*p* < 0.05) and prenenolone (*p* < 0.01) levels in FF of PCOS group were significantly increased, compared to the normal group, and the progesterone levels (*p* < 0.05) were decreased in PCOS group. Increased mRNA levels of *CYP11A, CYP19A* and *HSD17B2* of exosomes were accompanied by the hormonal changes in FF. Correlation analysis showed that mRNA levels of *CYP11A* and *HSD17B2* were negatively correlated with percent of top-quality embryos and rate of embryos develop to blastocyst.

**Conclusion:**

Our results suggest that increased levels of estrogen and pregnenolone in follicular fluid may affect follicle development in PCOS patients, and the mechanism is partially related to *HSD17B1*, *CYP19A1* and *CYP11A1* expression change in FF exosomes.

**Supplementary Information:**

The online version contains supplementary material available at 10.1186/s12958-021-00749-6.

## Background 

Polycystic ovary syndrome (PCOS) is a common endocrine and metabolic disorder [1], affecting 5–20% females of reproductive age [2]. This syndrome is multi-factorial and heterogeneous with variable phenotypes, including hyperandrogenism, menstrual irregularity and polycystic ovarian morphology [3]. PCOS is the leading cause of anovulatory infertility and anovulation is the main characteristic. Follicular fluid (FF) contains factors responsible for oocyte maturation and ovulation [4]. Previous studies have shown that bioactive steroids, in particular androgens (testosterone, dihydrotestosterone), estrogens (estradiol, estrone) and progesterone in FF play important roles in regulating ovarian folliculogenesis and oocyte maturation [5]. Studies have found oocyte quality and outcomes of in-vitro fertilization (IVF) in PCOS are related with components changes in FF [6, 7], yet the mechanisms have not been fully elucidated.

In PCOS patients during assisted conception, the quality of oocyte and embryo and pregnancy outcomes inclined to be poorer, which may be linked to changes in the oocyte through the microenvironment of follicular fluid (FF) [8]. The FF of the dominant follicle can reflect the ovarian microenvironment to some extent [9, 10], which directly affects the oocyte quality and metabolism.

Several studies have analyzed follicular fluid steroid concentrations to determine whether these can predict IVF outcomes [11, 12]. Most have used steroid immunoassays, which are suboptimal when applied to biological fluids [13]. Therefore, we sought to investigate follicular fluid steroid profiles using liquid chromatography-tandem mass spectrometry (LC-MS/MS) profiling. In our previous study [14], we have developed a multi-analyte LC-MS/MS method for the profiling of 20 steroids, including progesterone, estradiol, estrone, androstenedione, testosterone, dihydrotestosterone and most of these intermediates. The aim of this study was to profile 20 steroids in the fluid from the dominant follicle in women undergoing IVF stimulation by LC-MS/ MS method, and to determine whether such highly specific steroid measurements improved prediction of IVF outcomes.

Recently, an increasing number of studies have shown that exosomes are present in FF and act as message transmitters in intercellular communication, which can transfer a variety of substance such as proteins, lipids, miRNAs, and circRNAs [15–19]. Exosomes are 30–200 nm in diameter with small membrane-enclosed vesicles, which are secreted by living cells under normal or pathophysiological circumstances [20]. Equine, bovine and human studies have all demonstrated the presence of exosomes in FF [21–23]. Some studies uncovered the potential function of exosomes derived from FF as carriers of RNAs, miRNAs and proteins in steroidogenesis, follicular development and other pathological conditions [23]. Ovarian steroidogenesis patterns are attributed mainly to the altered expression of key enzymes in the steroidogenic pathway such as cholesterol side-chain cleavage enzyme cytochrome P450, 17α-hydroxylase, and 3β-hydroxysteroid dehydrogenase. Genes involved in hormone synthesis include *STAR, CYP11A, CYP19A, HSD17B1* and *HSD3B2.*

The primary aim of the current study was to determine different steroid profile of follicular fluid in PCOS women and non-PCOS women undergoing reproductive measures. We also try to explore the underlying mechanisms of estrogens excess in PCOS FF using exosome.

## Materials and methods

### Patient selection

Samples were collected from September 2019 to November 2019. Patients have given their written informed consent. The study was approved by the Ethical Committee of the Zhongshan Hospital, Fudan University (Shanghai, China), and carried out in compliance with the Population and Family Planning Law of the People’s Republic of China (The ethical approval number: B2018–244).

The diagnosis of PCOS has based on the 2003 Rotterdam diagnostic criteria which require two of the following three manifestations: (1) oligo- and/or anovulation, (2) clinical and/or biochemical evidence of hyperandrogenism, and (3) polycystic ovaries on ultrasound examination (at least 10 follicles 2–9 mm in size or volume of the ovary greater than 10 mL) [3]. Ten patients were selected as the PCOS group who require the three manifestations at the same time and underwent in vitro fertilization (IVF) with intracytoplasmic injection (ICSI) at Reproductive Center, Zhongshan Hospital, Fudan University. Another ten women who underwent IVF treatment with an indication of male factor infertility during the same period were selected as the control group. All the recruited patients were under 36 years old and had a normal BMI range from 18.8 to 25 kg/m^2^. All patients received the antagonist stimulation protocol as reference [24].

### Clinical examination

The BMI values, hormonal, biochemical and ultrasonographic parameters of the control and PCOS patients were documented. BMI (kg/m^2^) was calculated as the ratio of the weight (kg) to the square of the height (m^2^). Patients received a standard in vitro fertilization (IVF) flexible-start antagonist stimulation protocol with exogenous FSH and human menopausal gonadotropins at individualized doses and underwent transvaginal ultrasound-guided follicle aspiration 36 h after human chorionic gonadotropin (hCG) and/or a GnRH agonist.

### Follicular fluid collection

The follicular fluid of the largest, first punctured follicle was collected during the oocyte retrieval procedure (transvaginal follicular aspiration). All the follicles we selected are surrounded by the cumulus-oocyte complexes. Each ovarian follicle was aspirated independently. The collected FF was checked for red blood cells. FF contaminated with red blood cells was excluded from the study.

### Assessment of oocyte and embryo quality

All oocytes retrieved from the FF samples were cultured and fertilized in a separate culture dish for evaluating the quality and embryo development of oocytes. Normal fertilization was defined by the presence of two pronuclei (2PN). A top-quality embryo was defined as an embryo with (i) seven or more blastomeres on day 3, (ii) 20% fragmentation or less on day 3, and (iii) no multinucleated blastomeres ever as described by Van Royen et al. [25]. On the fifth day of the culture, at the blastocyst stage, three criteria were taken into consideration: the development of inner cell mass (ICM), the appearance of trophectoderm (TE) and the expansion of the blastocyst cavity. Blastocyst Day 5–6 on was considered to be a fully expanded (Grade 4) through to a hatched (Grade 5) blastocyst with a high-quality ICM (Grade 1) and TE (Grade 1) [26].

### Steroid profiling in follicular fluid

Twenty steroids were measured in 200 μl of follicular fluid samples using LC-MS/MS. Details of the LC- MS/MS methods are presented in the reference [14]. The panel included progestogens (including mineralocorticoids and glucocorticoids), androgens and estrogens biosynthesized in steroid metabolic pathways. Because of the high concentrations of 17-OH pregnenolone, progesterone, estradiol and estrone, follicular fluid samples were run a second time diluted 100-fold with double-stripped, delipidated normal human plasma (blank matrix plasma, Product code: 1800–0058, SeraCare Life Sciences, MA, USA) to quantify those three steroids.

### Exosome isolation

The follicular fluid exosomes were isolated by ultracentrifugation. Follicular fluid was centrifuged at 300 g for 10 min to remove cells. The supernatant fluid was then centrifuged at 2000 g for 10 min at 4 °C to remove dead cells. The resultant supernatant fluid was transferred to an ultracentrifuge tube and centrifuged at 100000 g for 2 h. The pellet was suspended in PBS and filtered through a 0.22-μm filter, and then centrifuged at 100000 g for 2 h. The pellet was resuspended in 200 μL PBS and stored at − 80 °C.

### Transmission electron microscopy

A total of 20 μL of exosome suspension (5 μg/μL) was fixed on a continuous grid and then negatively stained with 2% uranyl acetate solution for 1 min and air-dried. The samples were observed by FEI Tecnai G2 spirit transmission electron microscope (FEITM) at an acceleration voltage of 120 kV.

### Nanoparticle tracking analysis

Nanoparticle tracking analysis (NTA) measurements were performed using a NanoSight NS300 instrument (Malvern Panalytical) with a 488-nm laser and sCMOS camera module (Malvern Panalytical). Measurements in flow mode were performed with a flow rate of 50, these flow measurements consisted of 3 measurements of 60 s, and the captured data were analysed using NTA 3.2 software.

### Western blot analysis

The concentration of proteins was quantified using the Pierce BCA Protein Assay Kit (Thermo Fisher Scientific) following the manufacturer’s instructions. Proteins were separated by 12% SDS-PAGE gels and transferred to PVDF membranes by gel electrophoresis and electroblotting, respectively. After blocking with 5% BSA, blots were probed with primary antibodies at 4 °C overnight. Then, membranes were washed and incubated with secondary antibodies. Ultimately, proteins were visualized using the enhanced chemiluminescence reagents (Thermo Fisher Scientific). CD9 antibody: CD9 (D8O1A) Rabbit mAb (#13174, Cell signaling technology); CD63 antibody: CD63 (D4I1X) Rabbit mAb (#55051, Cell signaling technology); TSG101 (ab125011, abcam); Calnexin (#2433, Cell signaling technology).

### RNA isolation and quantitative RT-PCR

Total RNA was extracted using TRIzol (Invitrogen), and RNA was then reverse-transcribed using SuperScript First-Strand cDNA System (Takara) according to the manufacturer’s instructions. Quantitative RT-PCR (qRT-PCR) was performed using the SYBR Green PCR master mix (Takara) and the StepOnePlus PCR system (Thermo Fisher Scientific) according to the manufacturer’s instructions. The house- keeping gene GAPDH was used as an endogenous control. The primer sequences are shown in Table S3.

### Statistical analysis

The Statistical Package for Social Sciences, version 21.0 (SPSS Inc., Chicago, IL, USA) was used for statistical analysis. Independent samples t-tests for normally distributed variables and Mann-Whitney U test for not-normally distributed variables were used to compare groups. The two-tailed unpaired t-test was used to evaluate the difference between PCOS and control group. Correlation analysis between FF steroid and other clinical and laboratory parameters were performed with the Pearson test for normally distributed variables and the Spearmen test for not normally distributed parameters. Normally distributed variables were presented as means and SEM and variables with skewed distribution were described as median and IQR. For all comparisons, statistical significance was defined by *p* < 0.05.

## Results

### Characteristics of the study population and outcomes of controlled ovarian stimulation

This study included ten PCOS patients and ten male-factor infertility patients. The demographic features, biochemical, and hormonal data of PCOS group and normal group are showed in Table 1. No statistically significant differences were found between the groups in relation to age, BMI, basal FSH, LH, estradiol and progesterone levels. Baseline testosterone was higher in PCOS women. Furthermore, more oocytes and less MII oocytes were retrieved in the PCOS women. The rate of retrieved top-quality embryos and embryos develop to blastocyst were significantly lower in women with PCOS. These data indicate that the quality of oocyte and embryo tend to be lower in PCOS patients during assisted conception, which may be linked to metabolism-induced changes in the oocyte through the microenvironment of follicular fluid (FF).

**Table 1:** Comparison of the clinical characteristics, endocrinological variables of PCOS patients and controls

	Control (*n* = 10)	PCOS (*n* = 10)	*P* value
Age (year)	30.60 ± 1.06	30.40 ± 1.09	NS
BMI (kg/m^2^)	20.73 ± 0.95	20.84 ± 0.94	NS
Basal serum LH (mIU/mL)	5.40 ± 0.65	6.37 ± 1.14	NS
Basal serum FSH (mIU/mL)	7.56 ± 0.89	7.14 ± 0.59	NS
Basal serum E2 (pmol/mL)	451.99 ± 316.91	708.7 ± 363.77	NS
Basal serum T (ng/ mL)	0.54 ± 0.09	1.20 ± 0.13	0.001
Basal serum P (nmol/L)	0.63 ± 0.12	0.75 ± 0.12	NS
Number of oocytes retrieved	11.1 ± 1.36	19.2 ± 1.98	0.003
MII oocytes (%)	89.1 ± 1.99	72.4 ± 16.39	0.022
Rate of fertilization (%)	77.3 ± 3.92	69.9 ± 6.50	NS
Top-quality embryo (%)	63.5 ± 5.71	33.3 ± 2.88	0.001
Rate of embryos develop to blastocyst (%)	76.7 ± 8.51	23.1 ± 7.08	0.001

Abbreviations: *BMI* Body mass index, *FF* Follicular fluid, *FSH* Follicle stimulating hormone, *LH* Luteinizing hormone, *E2* Estradiol, *T* Testosterone, *PROG* Progesterone, *Ret. oocyte* Number of oocytes retrieved, *MII oocytes* Number of metaphase II oocytes, *NS* Not significant. Data are expressed as mean ± SEM. Differences between groups were considered significant for *p* ≤ 0.05

### FF steroid profiles in PCOS and normal control participants

**Fig. 1:**
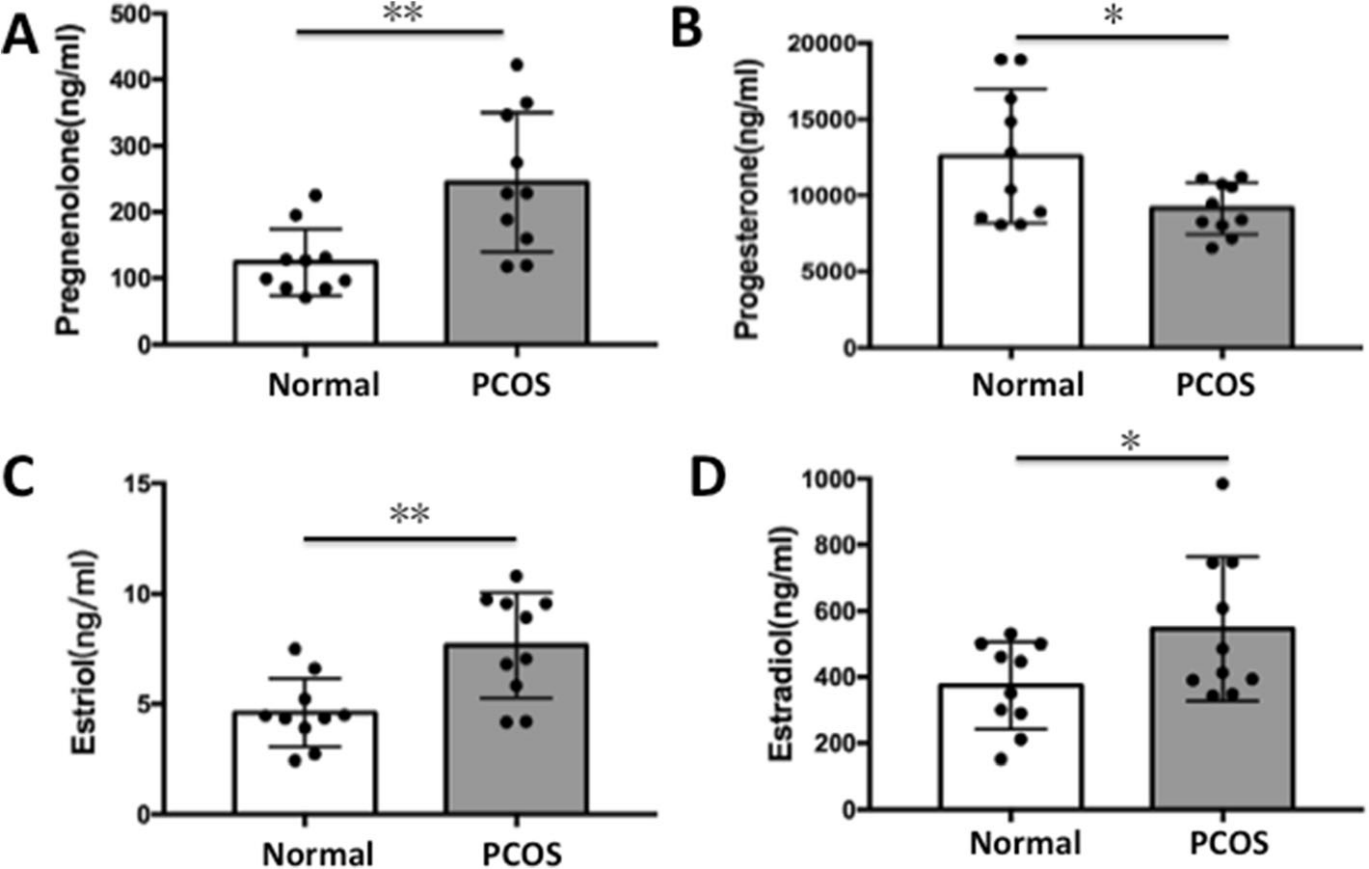
Distribution of steroid concentrations in follicular fluid from PCOS patients and controls. (**a**) Pregenolone; (**b**) Progesterone; (**c**) Estriol; (**d**) Estradiol. Data are presented as mean ± SD. *p < 0.05, **p < 0.01

To evaluate the possibility of using key reproductive hormones as potential indicators of oocytes development, 20 steroids were quantified and compared between PCOS patients and controls by LC-MS/MS. The data displayed in Table S1 show that the control group had lower pregnenolone levels in follicular fluid (mean, 123.91 ± 15.93 ng/mL) than the PCOS women (mean, 245.04 ± 33.40 ng/ml) and the differences were statistically significant (*p* = 0.006). Progesterone levels in follicular fluid were higher in normal group (mean, 12,590 ± 1393.59 ng/ml) than they were in PCOS (mean, 9153.38 ± 542.39 ng/ml) (*p* = 0.041). FF estriol levels were significantly increased (*P* = 0.003) in PCOS group (7.66 ± 0.76 ng/ml) compared to the normal group (4.60 ± 0.49 ng/ml) (Table S1, Fig. 1). Similarly, FF estradiol levels were significantly higher (*P* = 0.047) in PCOS group (546.32 ± 68.95 ng/ml) compared to normal group (374.38 ± 41.92 ng/ml) (Fig. 1).

### Correlation of follicular fluid estrogen and progestogen level with embryo quality

**Fig. 2:**
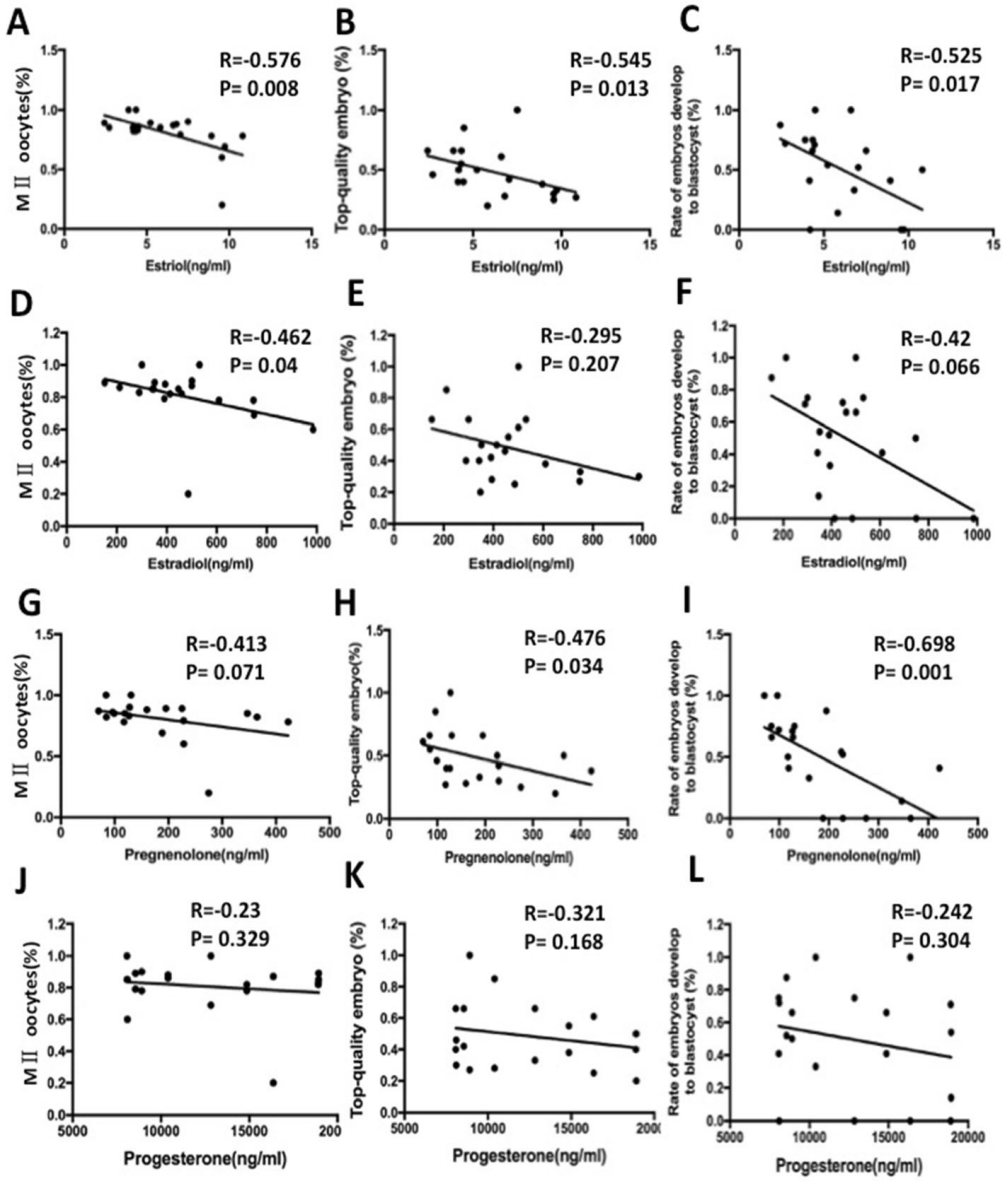
Correlation of follicular fluid estriol, estradiol, pregnenolone and progesterone level with embryo quality. (**a** & **d** & **g** & **j**) Correlation of follicular fluid estriol, estradiol, pregnenolone and progesterone level with MII oocytes punctured; (**b** & **e** & **h** & **k**) Correlation of follicular fluid estriol, estradiol, pregnenolone and progesterone level with percent of top-quality embryos; (**c** & **f** & **i** & **l**) Correlation of follicular fluid estriol, estradiol, pregnenolone and progesterone level with rate of embryos develop to blastocyst. Values are significant at *p* < 0.05

We further elucidated whether the estrogen and progestogen level predicted the outcome of ovarian stimulation and embryo quality. Our results showed that, with increasing FF estriol levels, significantly less MII oocytes (*r* = − 0.576, *p* = 0.008) (Fig. 2a) were obtained in PCOS women, and tended to have a reduced chance to develop into a morphological top-quality embryo (*r* = − 0.545, *p* = 0.013) (Fig. 2b). Moreover, follicular fluid estriol levels were negatively correlated with rate of blastocysts (*r* = − 0.525, *p* = 0.017) (Fig. 2c). Increased estradiol levels in follicular fluid were associated with a lower rate of MII oocytes (*r* = − 0.462, *p* = 0.04) (Fig. 2d). Furthermore, pearson correlation analysis showed that FF levels of pregnenolone were negatively and significantly correlated with top-quality embryo (*r* = − 0.476, *p* = 0.034) (Fig. 2h) and rate of embryos develop to blastocysts (*r* = − 0.698, *p* = 0.001) (Fig. 2i). Progesterone level was not significantly related with embryo quality (Fig. 2j & k & l). This may be the evidence of the immaturity of follicles as well as negative effects of estriol and estradiol on folliculogenesis and oocyte quality in PCOS patients.

### *CYP11A1, CYP19A1* and *HSD17b1* mRNA expression in FF exosomes

**Fig. 3:**
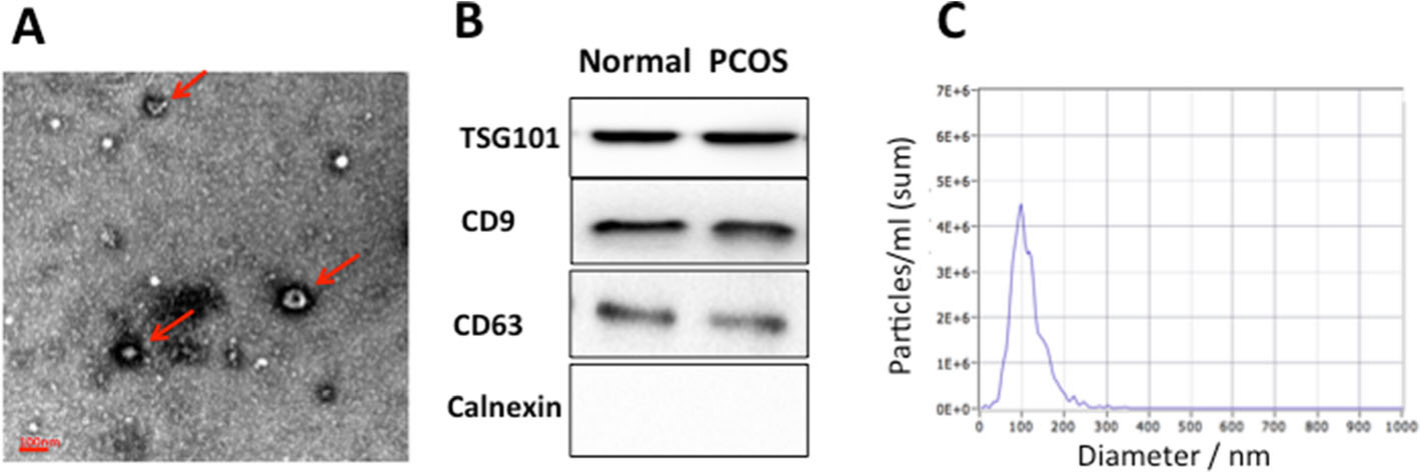
Quality control of extracted follicular fluid exosomes. (**a**) Electron microscopic images of extracted exosomes revealed cup-shaped structures with a diameter of about 30–150 nm. Scale bar: 100 nm. (**b**) Western blotting revealed CD9, CD63 and TSG101 proteins in exosome samples. (**c**) Nanoparticle tracking analysis (NTA) revealed the diameter of isolated extracellular vesicles (EVs) is consistent with that of exosomes

Human FF exosomes were characterized with regard to their morphology, diameter and the presence of exosome-enriched protein markers. Electron microscopic images (Fig. 3a) showed that extracted extracellular vesicles (EVs) were cup-shaped structures with a diameter of about 30–150 nm. Moreover, the presence of the exosome markers CD63, CD9 and TSG101 proteins were confirmed by western blot (Fig. 3b). The size distribution measured by NTA also showed a typical profile of exosomes (Fig. 3c). TEM, NTA and Western blot results were consistent with the characteristics of exosomes.

**Fig. 4:**
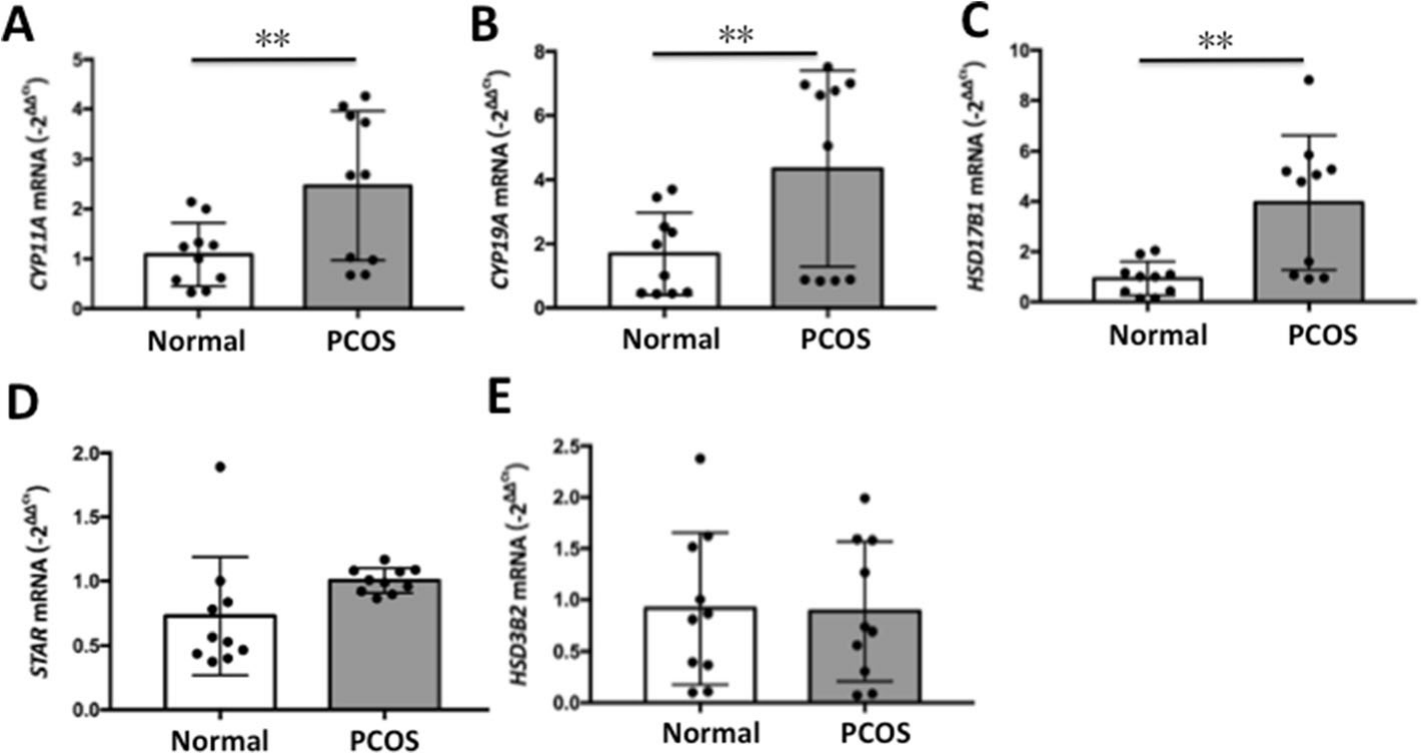
*CYP11A1, CYP19A1, HSD17b1, STAR* and *HSD3b2* mRNA expression in follicular fluid exosomes. (**a**) *CYP11A*, CYP11A cytochrome P450 side-chain cleavage enzyme; (**b**) *CYP19A*, CYP19A cytochrome P450 side-chain cleavage enzyme; (**c**) *HSD17B1*, hydroxysteroid 17beta dehydrogenase 1; (**d**) *STAR*, steroidogenic acute regulatory protein. (**e**) *HSD3B2*, hydroxysteroid 3beta dehydrogenase 2. Data are presented as mean±SD; *p < 0.05, **p < 0.01

The mRNA expression levels of these genes from the FF exosomes were examined in our study, and the results showed significantly increased *CYP11A, CYP19A* and *HSD17b1* mRNA expression in the PCOS group (Fig. 4), indicating that the increase in E2 was associated with increased expression of key genes involved in hormone synthesis. Whereas *STAR* and *HSD3b2* mRNA expression were not affected (*p* > 0.05). *CYP19A1* encodes cytochrome P450 aromatase that converts androgens to 17β estradiol [27]. *HSD17B1* is expressed in the syncytiotrophoblast cells of the placenta [28] and ovarian granulosa cells [29], and both of these cell types are involved in de novo estrogen biosynthesis. *CYP11A1* is the gene encoding for the key enzyme in metabolism of cholesterol to pregnenolone. The increased mRNA expression on exosome in FF is consistent to the increased pregnenolone level in FF in PCOS patients. The increases in the expression levels of *HSD17B2, CYP11A* and *CYP19A* were accompanied by the changes in hormonal levels in FF, indicating a condition shifting from the progesterogenic follicles to estrogenic follicles in PCOS patients.

### Correlation of *CYP11A, CYP19A* and *HSD17b1* mRNA level with hormone in FF and embryo quality

**Fig. 5:**
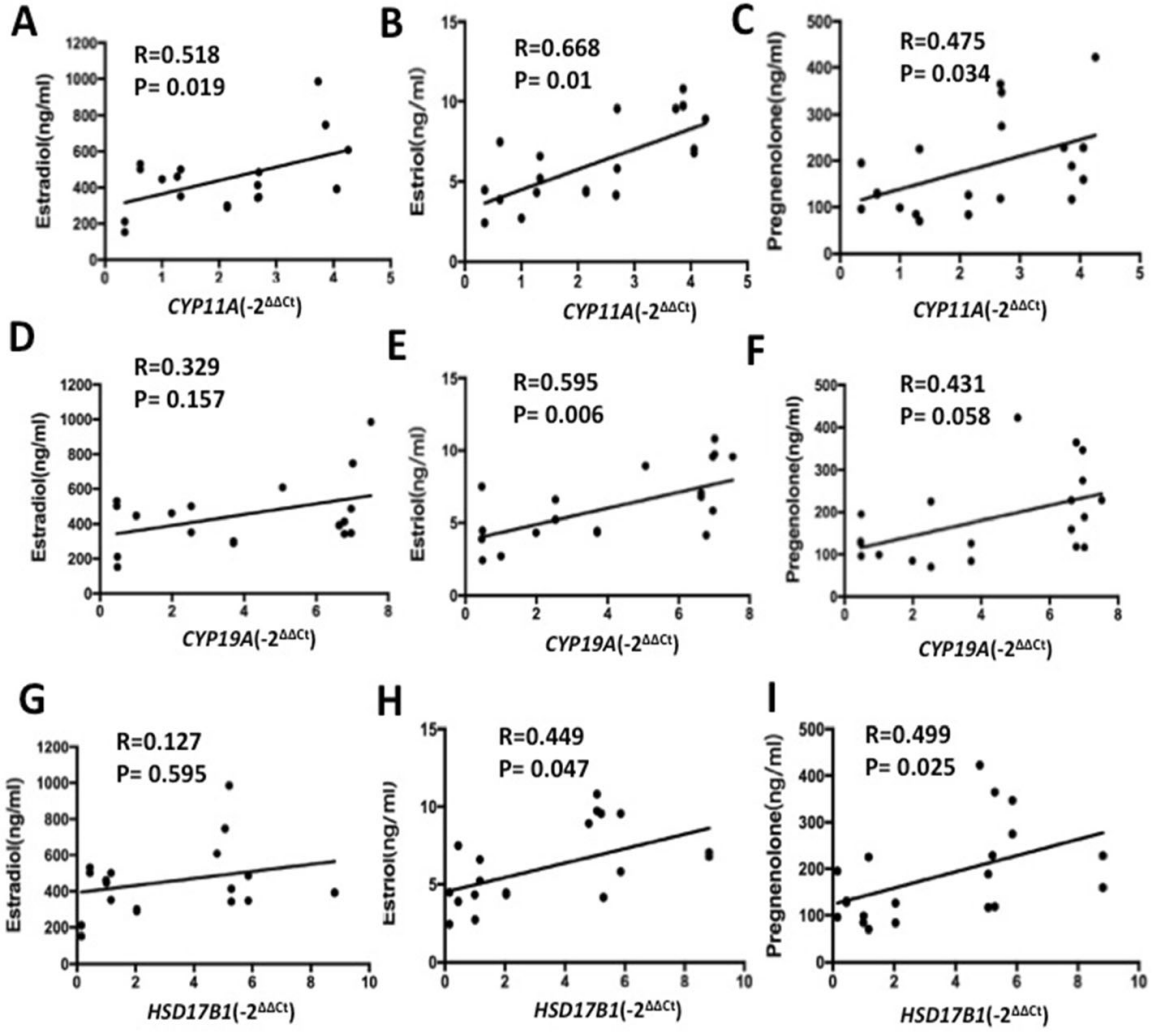
Correlations between the *CYP11A, CYP19A* and *HSD17b1* mRNA level in exosomes with estradiol (**a** & **d** & **g**), estriol (**b** & **e** & **h**) and prenenolone(**c** & **f** & **i**). Values are significant at *p* < 0.05

We further elucidated whether *CYP11A, CYP19A* and *HSD17b1* mRNA level predicted the hormone in FF and the outcome of ovarian stimulation and embryo quality. Pearson correlation analysis (Fig. 5) showed that *CYP11A* mRNA level was positively and significantly correlated with estradiol, estriol and pregnenolone levels. The elevated *CYP19A* mRNA level was coraleted with estradiol. There is a positive correlation between *HSD17b1* mRNA in exosomes from FF and estradiol and pregnenolone was significant.

**Fig. 6:**
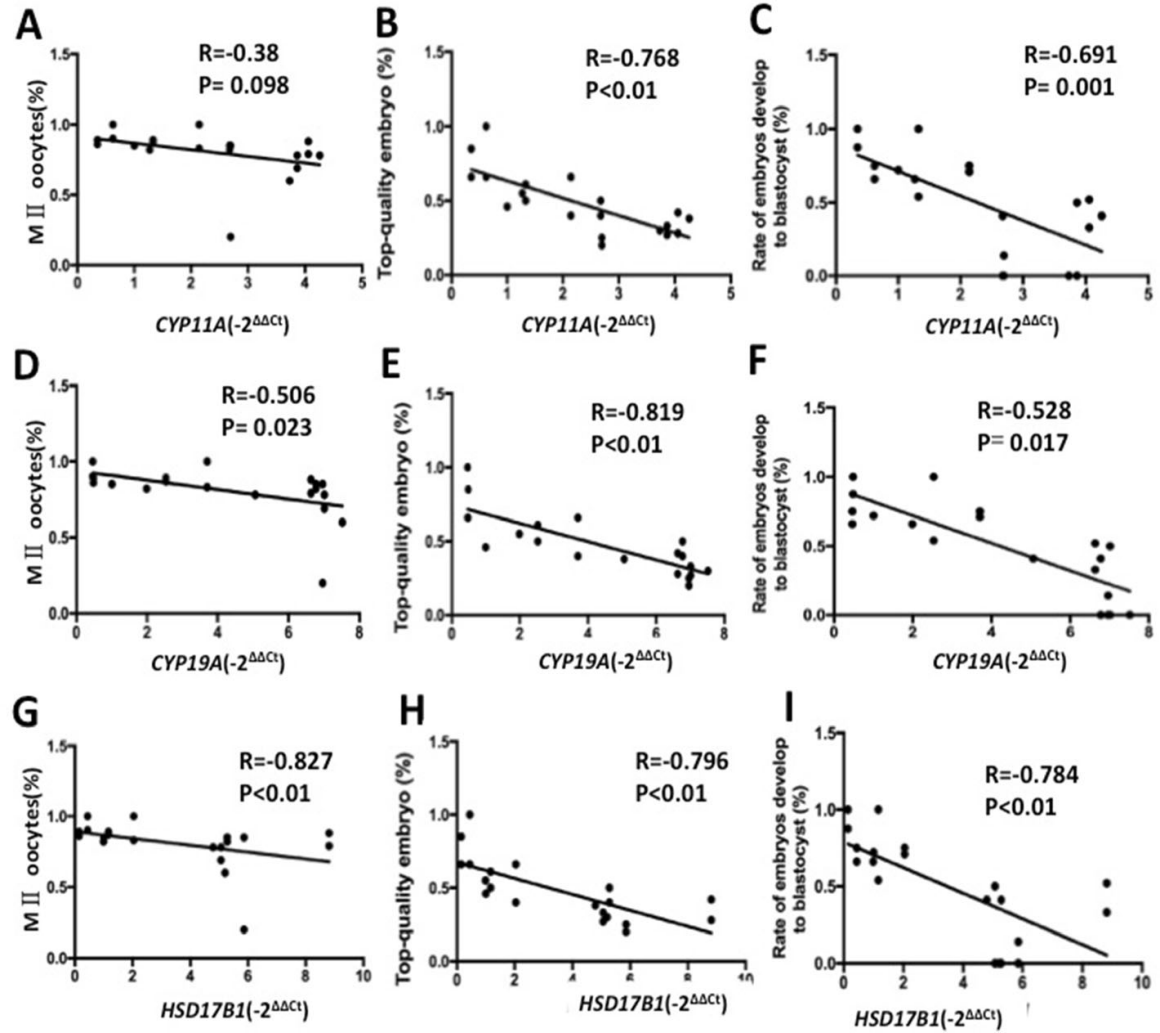
Correlation of *CYP11A1, CYP19A1* and *HSD17b1* mRNA level in follicular fluid exosomes with embryo quality. (**a** & **d** & **g**) Correlation of *CYP11A1, CYP19A1* and *HSD17b1* mRNA level in follicular fluid exosomes with MII oocytes punctured; (**b** & **e** & **h**) Correlation of *CYP11A1, CYP19A1* and *HSD17b1* mRNA level in follicular fluid exosomes with percent of top-quality embryos; (**c** & **f** & **i**) Correlation of *CYP11A1, CYP19A1* and *HSD17b1* mRNA level in follicular fluid exosomes with percent of rate of embryos develop to blastocyst. Values are significant at *p* < 0.05

Furthermore, pearson correlation analysis showed that exosomal *CYP11A* mRNA level were negatively and significantly correlated with top-quality embryo (*r* = − 0.768, *p* < 0.01) (Fig. 6b) and rate of embryos develop to blastocysts (*r* = − 0.691, *p* < 0.01) (Fig. 6c). Our results also showed that, with increasing exosomal *CYP19A* mRNA levels, significantly less MII oocytes (*r* = − 0.506, *p* = 0.023) (Fig. 6d) were obtained in women, and tended to have a reduced chance to develop into a morphological top-quality embryo (*r* = − 0.819, *p* < 0.01) (Fig. 6e). Moreover, exosomal *CYP19A* mRNA levels were negatively correlated with rate of blastocysts (*r* = − 0.528, *p* = 0.017) (Fig. 6f). Increased *HSD17b1* mRNA levels in follicular fluid were associated with a lower rate of top-quality embryo (*r* = − 0.796, *p* < 0.01) (Fig. 6h) and blastocysts (*r* = − 0.784, *p* < 0.01) (Fig. 6i). There is a negative correlation between *CYP11A, CYP19A* and *HSD17b1* mRNA level in exosomes in follicular fluid and oocyte quality.

## Discussion

Previous studies have found that PCOS women having IVF presents multiple challenges ranging from a poor to an exaggerated response, poor egg to follicle ratio, poor fertilisation, poor blastocyst conversion and ovarian hyperstimulation syndrome [30]. Despite limited number of patients, we can also find the decreased MII oocytes, decreased top-quality embryo and the rate of embryos develop to blastocyst. For PCOS patients, more emphasis has been placed on obtaining the high-quality embryos in order to improve the pregnancy rate.

In the present study, we measured the concentrations of twenty steroids in follicular fluid by sensitive and specific multi-analyze LC-MS/MS. Most studies measured one or more steroids by immunoassay for each steroid. Steroid immunoassays have specific limitations in the application of biological fluids [31, 32]. Therefore, there may be differences between the study of the content of steroids in follicular fluid and its relationship with the outcome of in vitro fertilization. In addition, each immunoassay requires a separate sample, which requires a larger sample size by pooling follicular fluid. This makes it impossible to provide a comprehensive analysis of steroids in a single follicle. Therefore, in this study, we extended these studies by using multi-analyze steroid LC-MS/MS for bioactive steroids in 200 μl matched samples of follicular fluid.

Human follicular fluid is a complex biological fluid, which plays an important role in the development of oocyte microenvironment. Previous studies have found various factors in FF was significantly different in PCOS patients compared with non-PCOS, such as homocysteine level [33], platelet factor 4 [34], endorphin [35], arachidonic Acid [36]. More and more evidence suggests that sex hormones in follicular fluid (FF) may play an important role in regulating the developmental potential of oocytes. Through our study, we found differences in the levels of estrogen and progesterone in follicular fluid of patients with PCOS. Zewn Li et al. [37] also found the estradiol concentrations in follicular fluid were higher in PCOS subjects compared with controls, which is consistent with our results. In women, the dominant follicle is the principle site of estrogen production during the follicular development [38]. Estrogen synthesized by granulosa cells generally has been assigned an autocrine function, regulating cell replication and cytodifferentiation in response to endocrine stimulation by FSH and LH [39].

Although biomarkers of hormones and other molecules have been evaluated in the FF [40], there are few consistent results in different literatures. This lack of consensus can be related to the criteria used in these studies of FF samples; differences in methods for measuring analytes, patient age, body mass index (BMI), infertility or other disease diagnosis. Another reason may be linked to the human ability with ART procedure or the psychophysiological characteristics of each woman (or couple). Many of these aspects affect the content of follicle hormones and reflect the oocyte and embryo quality. The dominant follicle is the largest follicle of multiple follicles, which is the clearest and free from contamination (especially blood cells). Therefore, we assure that the results of the hormonal measurement are sound. We believe that the results are conclusive even though the sample size of this study is limited.

EV-encapsulated mRNAs are shielded from degradation and remarkably stable in biological fluids. Compared with the analysis of total RNA, the study of EV-encapsulated mRNAs has key biological advantages. Investigating EV-encapsulated mRNAs also has key biological advantages over analysis of total mRNAs. EV mRNAs are actively released by living cells, which is a better representation of the active ways of communication between cells and tissues. However, the total mRNAs in human body fluids can also be released from apoptotic cells or cell debris. Steroidogenic granulosa and theca cells produce estrogens by stimulating synthesis of steroidogenic enzyme messenger RNAs under the control of gonadotropin. The expression of *HSD17B1* and P450 aromatase (*CYP19A1*) is related with follicular differentiation and follicular E2 concentration.

Currently, it is impossible to accurately predetermine the developmental potential of oocytes before in vitro embryo production. Such a test would be able to reduce the production of reduntant embryos, thereby overcoming the ethical, legal, and storage impact of current human assisted reproduction practices among the PCOS patients. It is currently recommended that a minimum number of 8–10 MII oocytes undergo vitrification to obtain a reasonable chance of pregnancy [41]. It is possible to provide an individualized treatment plan for PCOS patients to retrieve the optimal number of high-quality oocytes.

## Conclusions

Our results indicate that in infertile PCOS patients, the steroid profile in follicular fluid was obviously altered. Increased levels of estrogen and pregnenolone in follicular fluid may affect follicle development in PCOS patients, and the mechanism may be related to *HSD17B1, CYP19A1* and *CYP11A1* expression change in FF exosomes, which encode enzyme-induced abnormalities in steroidogenesis. *HSD17B1, CYP19A1* and *CYP11A1* mRNA expression change in FF exosomes can be innovative markers of oocyte competence in clinical practices. The combination of follicular fluid analysis and routine morphological assessment can provide a more accurate and sensitive method for determining the embryonic developmental ability of PCOS patients.

## Supplementary Information

**Additional file 1 Table S1.** Follicular fluid steroid levels in PCOS patients and controls. Abbreviations: 17-OHP: 17-hydroxypregnenolone; 21-OHP: 21-hydroxy pregnenolone; DHT: Dihydrotestosterone; DHEA, dehydroepiandrosterone; A4: Androstenedione. Data are expressed as mean ± SEM. Independent samples t-tests for normally distributed variables and Mann-Whitney U test for not-normally distributed variables (* are not-normally distributed variables) were used to compare groups. Differences between groups were considered significant for *p* ≤ 0.05. **Table S2.** Correlation coefficients between follicular fluid steroid levels and measured parameters. Values are significant at *p* < 0.05. **Table S3.** The primer sequences for RT-PCR.

## Data Availability

The data that support the study are available upon reasonable request to the corresponding author.

## Abbreviations

PCOS: Polycystic ovary syndrome
FF: Follicular fluid
LC-MS/MS: Liquid chromatography-tandem mass spectrometry
IVF: In vitro fertilization
ICSI: Intracytoplasmic injection
hCG: Human chorionic gonadotropin
2PN: Two pronuclei
ICM: Inner cell mass
TE: Trophectoderm
FEITM: FEI Tecnai G2 spirit transmission electron microscope
NTA: Nanoparticle tracking analysis
EVs: Extracellular vesicles
