# Supplementary material for: Correlation between steroid levels in follicular fluid and hormone synthesis related substances in its exosomes and embryo quality in patients with polycystic ovary syndrome
Source: Reprod Biol Endocrinol. 2021 May 17;19:74. doi: 10.1186/s12958-021-00749-6 (PMC8127216; doi:10.1186/s12958-021-00749-6)
Supplement: Supplementary file 1 — Additional file 1 Table S1. Follicular fluid steroid levels in PCOS patients and controls. Abbreviations: 17-OHP: 17-hydroxypregnenolone; 21-OHP: 21-hydroxy pregnenolone; DHT: Dihydrotestosterone; DHEA, dehydroepiandrosterone; A4: Androstenedione. Data are expressed as mean ± SEM. Independent samples t-tests for normally distributed variables and Mann-Whitney U test for not-normally distributed variables (* are not-normally distributed variables) were used to compare groups. Differences between groups were considered significant for p ≤ 0.05. Table S2. Correlation coefficients between follicular fluid steroid levels and measured parameters. Values are significant at p < 0.05. Table S3. The primer sequences for RT-PCR. [file 12958_2021_749_MOESM1_ESM.docx]

Supplemental material

Table S1

Follicular fluid steroid levels in PCOS patients and controls.

|  | Percent detectable samples | Control（n=10） | PCOS（n=10） | P value |
| --- | --- | --- | --- | --- |
| Pregnenolone*(ng/ml) | 100 | 123.91±15.93 | 245.04±33.40 | 0.006 |
| Progesterone(ng/ml) | 100 | 12590.98±1393.59 | 9153.38±542.39 | 0.041 |
| 17-OH Pregnenolone(ng/ml) | 100 | 52.86±2.44 | 60.18±5.01 | 0.21 |
| 17-OHP(ng/ml) | 100 | 1191.92±72.45 | 1151.49±130.16 | 0.79 |
| 21-ohP(ng/ml) | 100 | 17.06±1.96 | 16.31±1.60 | 0.77 |
| DHEA(ng/ml) | 100 | 22.94±2.29 | 20.05±5.75 | 0.65 |
| Testosterone*(ng/ml) | 100 | 0.15±0.68 | 0.14±0.39 | 0.91 |
| A4*(ng/ml) | 100 | 3.10±1.03 | 3.5±1.06 | 0.79 |
| DHT*(ng/ml) | 100 | 0.23±0.09 | 0.34±0.08 | 0.34 |
| Estriol(ng/ml) | 100 | 4.60±0.49 | 7.66±0.76 | 0.003 |
| Estrone(ng/ml) | 100 | 71.09±12.56 | 98.84±14.92 | 0.17 |
| Estradiol(ng/ml) | 100 | 374.38±41.92 | 546.32±68.95 | 0.047 |
| Corticosterone(ng/ml) | 100 | 2.13±0.16 | 2.36±0.14 | 0.31 |
| Cortisone(ng/ml) | 100 | 13.30±1.12 | 15.60±2.02 | 0.33 |
| Cortisol(ng/ml) | 100 | 53.68±5.53 | 58.98±6.17 | 0.53 |
| ALD*(ng/ml) | 100 | 1.36±0.23 | 0.08±0.12 | 0.07 |
| 11-deoxycortisol *(ng/ml) | 100 | 0.04±0.14 | 0.90±0.09 | 0.15 |
| 21-deoxycortisol* (ng/ml) | 80 | 0.04±0.01 | 0.12±0.003 | 0.09 |
| Melatonin*(ng/ml) | 100 | 0.02±0.004 | 0.03±0.008 | 0.24 |
| Dexamethasone*(ng/ml) | 55 | 0.17±0.006 | 0.21±0.005 | 0.59 |

Abbreviations: 17-OHP: 17-hydroxypregnenolone; 21-OHP: 21-hydroxy pregnenolone; DHT: Dihydrotestosterone; DHEA, dehydroepiandrosterone; A4: Androstenedione. Data are expressed as mean±SEM. Independent samples t-tests for normally distributed variables and Mann-Whitney U test for not-normally distributed variables (* are not-normally distributed variables) were used to compare groups. Differences between groups were considered significant for p ≤ 0.05.

Table S2: Correlation coefficients between follicular fluid steroid levels and measured parameters.

|  | Pregnenolone | | Progesterone | | Estriol | | Estradiol | |
| --- | --- | --- | --- | --- | --- | --- | --- | --- |
|  | R | P-value | R | P-value | R | P-value | R | P-value |
| Age (year) | -0.034 | 0.888 | -0.082 | 0.732 | -0.001 | 0.997 | -0.297 | 0.203 |
| BMI (kg/m^2^) | -0.074 | 0.756 | -0.149 | 0.532 | -0.134 | 0.573 | 0.171 | 0.472 |
| Basal serum LH (mIU/mL) | 0.063 | 0.79 | 0.056 | 0.815 | 0.113 | 0.637 | -0.002 | 0.995 |
| Basal serum FSH (mIU/mL) | 0.09 | 0.706 | -0.044 | 0.854 | 0.281 | 0.23 | -0.228 | 0.333 |
| Basal serum E2 (pmol/mL) | 0.299 | 0.201 | -0.122 | 0.609 | 0.081 | 0.733 | -0.32 | 0.169 |
| Basal serum T (ng/ mL) | 0.49 | 0.028 | 0.285 | 0.223 | -0.277 | 0.238 | -0.298 | 0.202 |
| Basal serum P (nmol/L) | 0.235 | 0.319 | -0.479 | 0.033 | 0.532 | 0.016 | 0.316 | 0.175 |
| Number of oocytes retrieved | 0.276 | 0.238 | -0.417 | 0.068 | 0.389 | 0.09 | 0.513 | 0.021 |
| MⅡ oocytes (%) | -0.413 | 0.071 | 0.23 | 0.329 | -0.576 | 0.008 | -0.462 | 0.04 |
| Rate of fertilization (%) | -0.05 | 0.833 | -0.06 | 0.803 | -0.184 | 0.438 | -0.035 | 0.882 |
| Top-quality embryo (%) | -0.476 | 0.034 | 0.321 | 0.168 | -0.545 | 0.013 | -0.295 | 0.207 |
| Rate of embryos develop to blastocyst (%) | -0.698 | 0.001 | 0.242 | 0.304 | -0.525 | 0.017 | -0.42 | 0.066 |

Values are significant at p < 0.05.

Table S3: The primer sequences for RT-PCR

| **Gene** | **Sense primer (5'-3')** | **Antisense primers (5'-3')** |
| --- | --- | --- |
| *CYP11A1* | TGGTGACAATGGCTGGCTAA | GAGGAATCGTTCTGGGTTGG |
| *STAR* | ATTCAAGCTGTGCGCTGGGAGC | TGGCCATCACAGCCTGTTGCC |
| *3βHSD2* | GTCATCCACACCGCCTGTAT | CACAGGCCTCCAACAGTAGC |
| *CYP19* | TTGGAAATGCTGAACCCGATAC | GCCAGTGAGGAGCAGGAC |
| *17βHSD* | TTCATGGAGAAGGTGTTGG | AAGACTTGCTTGCTGTGG |
| *Human 18S rRNA* | cagccacccgagattgagca | tagtagcgacgggcggtgtg |
